# Preventing Premature Family Maladjustment: Protocol for a Multidisciplinary eHealth Study on Preterm Parents’ Well-Being

**DOI:** 10.2196/63483

**Published:** 2025-03-18

**Authors:** Alessandra Decataldo, Federico Paleardi, Giacomo Lauritano, Maria Francesca Figlino, Concetta Russo, Mino Novello, Brunella Fiore, Giulia Ciuffo, Chiara Ionio

**Affiliations:** 1 Dipartimento di Sociologia e Ricerca Sociale Università degli Studi Milano-Bicocca Milano Italy; 2 Dipartimento di Psicologia Università Cattolica del Sacro Cuore Milano Italy; 3 Dipartimento di Psicologia, Unità di Ricerca sulla Psicologia del Trauma, CRIDEE Università Cattolica del Sacro Cuore Milano Italy

**Keywords:** preterm birth, parental well-being, sociology of health, digital-based monitoring, mixed methods research, eHealth

## Abstract

**Background:**

The consequences of preterm birth extend beyond the clinical conditions of the newborn, profoundly impacting the functioning and well-being of families. Parents of preterm infants often describe the experience of preterm birth and subsequent admission to the neonatal intensive care unit (NICU) as a disruptive event in their lives, triggering feelings of guilt, helplessness, and fear. Although various research examines changes in parents’ well-being and perception of self-efficacy during the stay in the NICU, there is a lack of research analyzing what happens in the transition phase at home after the baby’s discharge. Recently, scholars have advocated for the use of web-based support programs to monitor and prevent preterm family maladjustment and assist parents.

**Objective:**

This interdisciplinary research will develop a sociopsychological model focused on assessing the well-being of parents of premature infants during and after their stay in a NICU. Specifically, the study aims to (1) monitor the mental health of parents of premature infants both at the time of the child’s discharge from the NICU and in the first 6 months after discharge to prevent family maladjustment, (2) deepen our understanding of the role of digital tools in monitoring and supporting preterm parents’ well-being, and (3) study the potential impact of the relationship with health care professionals on the overall well-being of parents.

**Methods:**

This project combines mixed methods of social research and psychological support with an eHealth approach. The well-being of parents of premature infants will be assessed using validated scales administered through a questionnaire to parents of preterm infants within 6 NICUs at the time of the child’s discharge. Subsequently, a follow-up assessment of parental well-being will be implemented through the administration of the validated scales in a web application. In addition, an ethnographic phase will be conducted in the NICUs involving observation of the interaction between health care professionals and parents as well as narrative interviews with health care staff. Finally, interactions within the digital environment of the web application will be analyzed using a netnographic approach. We expect to shed light on the determinants of well-being among parents of premature infants in relation to varying levels of prematurity severity; sociodemographic characteristics such as gender, age, and socioeconomic status; and parental involvement in NICU care practices. With the follow-up phase via web application, this project also aims to prevent family maladjustment by providing psychological support and using an eHealth tool.

**Results:**

The results are expected by October 2025, the expiration date of the Project of Relevant National Interest.

**Conclusions:**

The eHealth Study on Preterm Parents’ Well-Being aims to improve preterm parents’ well-being and, indirectly, children’s health by reducing social costs. Furthermore, it promotes standardized neonatal care protocols, reducing regional disparities and strengthening collaboration between parents and health care staff.

**International Registered Report Identifier (IRRID):**

PRR1-10.2196/63483

## Introduction

### Background

The World Health Organization defines as preterm a birth that occurs before 37 weeks of gestation [1]—in Italy, approximately 6% of newborns are born before term [2]. Preterm birth is a multiproblematic event with 3 main consequences. First, it poses a medical risk to the newborns as many of these infants are in critical condition and can experience a range of substantially and potentially life-threatening medical complications. The second consequence concerns the economic and medical cost of caring for these babies given the advances in perinatal and neonatal care that have contributed to a substantial increase in the survival rate of these infants, particularly for extremely premature ones who, until a few decades ago, had no chance of surviving [3]. The third consequence is that the premature birth of a child and the subsequent admission to the neonatal intensive care unit (NICU) are distressing experiences for parents. Parental stress and parenting difficulties in the first years of the child’s life are well-known consequences [4]. Moreover, many longitudinal studies have described how prematurity negatively affects infants’ development [5,6], and evidence has been gathered supporting that parent well-being and infants’ long-term developmental outcomes are closely related [7]. Parents often describe premature birth and the subsequent hospitalization in a NICU as an emotional roller coaster and a disruptive life event [8]. An unexpected birth can trigger feelings such as guilt for not completing the pregnancy, helplessness, and fear of not being able to protect one’s child [6]. Moreover, parent-infant separation once admitted to the NICU represents a major source of stress for both parents and their infants [9]. Prematurity affects not just the child and parents as individuals (eg, by delaying the exploration of the parental role) but also the family triad (and potential siblings) as it hinders the initiation of mutual understanding and the consolidation of affectionate and meaningful relationships. Indeed, the hospital environment limits the parent-child relationship, which primarily develops through the incubator’s windows [10]. These restrictions, while serving to protect infants from pathogens, complicate the interaction between parents and newborn, making it even more challenging to activate caregiving modes necessary for the infant’s psychological development, a task primarily undertaken by medical staff for clinical reasons [11]. Another important factor to consider, highlighted by Russo et al [12], is that the sociodemographic characteristics of parents act as independent stress predictors, with gender, occupational status, and age particularly playing a role in the levels of stress and depression among parents of hospitalized children. Furthermore, few studies have investigated fathers’ experiences in these particular circumstances [6]. In Italy, especially during the COVID-19 pandemic, many NICUs applied restrictions regarding the frequency and time of parents’ access, and fathers are usually less involved than mothers in practices such as skin-to-skin contact and kangaroo care, managing to start building a relationship with their babies only after discharge. As researchers have pointed out [13], it is important to consider the characteristics and points of view of both mothers and fathers as both parents are at risk after preterm births. Research suggests how parents’ negative feelings can be reduced by giving them value as actors who can make decisions for the newborn [14]. The Family Integrated Care (FICare) model represents an example of a way to address the need for humanization and parent participation in health care for high-risk newborns admitted to the NICUs. Through this approach, parents have the opportunity to become increasingly engaged in their responsibilities, and their active involvement in care is a prerequisite for the consolidation of parenting skills, which are essential to cope with the separation through the NICU [15]. However, a recent study has pointed out that, although most units in different European countries report a neonatal care policy that encourages parents to take part in the care of their infants, parental involvement is still generally limited in Italy [6].

In addition, there is some evidence suggesting that the feasibility of implementing FICare with families of infants who are severely premature or critically ill remains a concern [16]. These cases often involve significant medical complexity, requiring intensive care, which may limit opportunities for parental involvement. Furthermore, the emotional and psychological toll of caring for an infant who is critically ill can hinder parents’ ability to participate actively in caregiving activities, highlighting the need for tailored support to address these challenges. Although various research examines changes in parents’ well-being and perception of self-efficacy during the stay in the NICU, there is a lack of research analyzing what happens in the delicate transition phase at home after the baby’s discharge [17]. Moreover, to the best of our knowledge, no studies have specifically examined how socioeconomic factors such as parental income or geographic distance from the NICU influence participation in FICare. This represents a gap in the literature as these factors may pose challenges for certain families. To fill this gap, the intervention and participatory project aim to give voice to preterm parents and their experiences. An interdisciplinary study to set neonatal practices and enhance families’ well-being (the Study on Preterm Parents’ Well-Being [ParWelB]) was designed by the Department of Sociology and Social Research of the University of Milano-Bicocca (principal investigator: AD) and funded by the Cariplo Foundation. Starting in May 2021, ParWelB has been promoting constructive communication and collaboration among and between caregivers and parents in the NICU.

Recent studies highlight the importance of fostering the parent-newborn bond, showing that strengthening this interaction benefits both parents—through reduced anxiety and depression—and children, who experience improved neurological outcomes [6]. Longitudinal research further suggests that parental stress and anxiety can impair bonding and family adjustment, with potential consequences for the development of preterm infants [5]. Parental self-efficacy and social support play a crucial role in adapting after an adverse situation such as preterm birth [18]. Scannel [19] recently suggested how interventions that target the development of parental self-efficacy and social support can strongly impact the sense of competence, satisfaction in the parenting role, and resilience of all members of the family. Furthermore, Ionio et al [20] highlight in their work that having more information on how parents perceive neonatal care and an understanding of their needs also after the discharge of their newborn may allow health care staff to identify parents at risk, plan early interventions to meet their needs, and promote family functioning. Given the importance of building parents’ self-efficacy, new neonatal practices (for upcoming preterm infants and parents) should be shaped based not only on the biomedical knowledge perspective but also on preterm parents’ values, lived experiences, and perspectives.

The transition from the NICU to the home is a pivotal and challenging period for parents of preterm infants. It involves adjusting from the highly structured and professionally supported NICU environment to taking on full caregiving responsibilities at home. This shift can heighten feelings of uncertainty and stress, particularly as parents face reduced access to health care professionals and must navigate complex medical or developmental needs independently.

Our study is an interdisciplinary research project that, building on the premises of the ParWelB project, strives to assess preterm parents’ well-being during and after the hospitalization in the NICU by combining mixed methods (using research strategies such as standardized questionnaires with internationally validated scales and ethnography) and psychological support through an innovative and technology-driven approach. Recently, scholars have championed the use of web- and app-based support programs to both monitor and prevent preterm family maladjustment and assist parents who struggle with transitioning to the home [21,22]. Furthermore, the work of Garfield et al [23] with infants with very low birth weight showed that parents who received an app-based support program improved parental self-efficacy and discharge preparedness compared with the control group. Therefore, the Bicocca research unit decided to team up with the Department of Psychology of the Catholic University of the Sacred Heart of Milan to design an eHealth (information and communications technology use to the advantage of human health) research project that assesses parents’ well-being, particularly at the crucial time of discharge and the first 6 months at home, offering support to parents who are more fragile. Parenting self-efficacy has been understood as the way in which parents perceive their ability to care for their child [24] and has been associated with positive parental outcomes [25]. The well-being of parents refers to the fact that, given that preterm birth is a highly stressful event for parents [26], considering the difficulties and uncertainties they experience during this acute phase is of significant importance to prevent family maladjustment [27]. Families’ involvement in the care of their high-risk neonates at NICUs represents the main axis of parent-partnered care initiatives. Each preterm parent develops a personal way to evaluate the situation based on the NICU lived experience. Specific characteristics may also influence the response to the hospitalization of one’s child. Significant effects have been identified in relation to gender (mothers and fathers seem to differ in responses and relation to certain measures) and to the degree of social support that parents have access to. The latter has been found to impact positively the resiliency factor associated with coping strategies of families who have a child with a chronic illness [28], and there is a great need for studies investigating its impact on preterm parents.

Thus, the main focus of this research project is to develop a sociopsychological model focusing on the assessment of preterm parents’ well-being during and after admission to a NICU with the use of eHealth.

### Objectives

Stemming from these premises, our research’s main aims could be synthesized as follows: (1) to monitor the mental health of parents of premature infants, with a plan for early intervention and a 6-month follow-up to prevent family maladjustment; (2) to stimulate eHealth growth by advancing knowledge on the role of digital-based tools in monitoring and supporting the well-being of parents whose infants have been hospitalized in the NICU for critical health situations (prematurity but also a wide spectrum of disabling or fatal diseases); and (3) to study the potential impact of the relationship with health care staff on the overall well-being of parents. In addition, our research aims to investigate potential differences in psychological well-being between parturients and nonparturients by also studying the impact of certain social characteristics (eg, nationality, educational level, employment status, and cultural enjoyment).

## Methods

### Ethical Considerations

Participation in the study will be on a strictly voluntary basis. According to the Declaration of Helsinki, respondents will receive written and oral information about the study and provide signed consent. They could withdraw consent at any time with no consequences for the future treatment of themselves or their infants. The eHealth ParWelB (e-ParWelB) project is financed by the Italian Ministry of University and Research with Next Generation EU and was approved in 2022 as a Project of Relevant National Interest (protocol 20225R7XB3). The project was submitted for approval to the ethics committees of the involved universities (University of Milano-Bicocca and Catholic University of the Sacred Heart of Milan) and, subsequently, to the 6 different territorial ethics committees to which the hospitals involved in the study are affiliated. The data are pseudoanonymized, meaning that each questionnaire is linked to a unique alphanumeric code that can be traced back to the user’s web application code. Protective measures include compliance with legal and ethical standards; restricted access; and security protocols to prevent unauthorized access, disclosure, modification, or destruction. Finally, the project will not provide specific compensation to participants.

### Recruitment

This study will be conducted simultaneously in 6 different Italian NICUs. Focusing on several units simultaneously has several advantages. First, it enables us to build a sample of satisfactory size within a reasonable time frame. Italy has a low birth rate, and therefore, concentrating on only 1 unit would pose a significant risk of obtaining an inadequate sample for any complex statistical analysis. In addition, the presence of various NICUs introduces a comparative dimension to the study. Previous research on neonatal intensive care [29] has shown that each unit has its own unique identity with different routines, idiosyncrasies, and decision-making processes. This identity is strongly influenced not only by the cultural context in which the unit is embedded but also by the administrative structure of the hospital and the legislative framework foreseen for neonatal care practice. This is particularly relevant in the Italian case, where the management of the public health system happens mainly at the regional level, with relevant differences between regions. Moreover, this study includes 5 NICUs from the public sector and 1 belonging to a private hospital, adding another possible layer of differences in the work culture of the units.

This research focuses mainly on the population of preterm parents—more specifically, parents of children born before 36+6 weeks of gestational age (GA) and hospitalized in intensive or subintensive care units for at least 10 days (critical threshold of hospitalization length). Considering the research objectives, it is crucial to distinguish infants according to the severity of prematurity because of the strong impact that the latter has on the health of the child and the duration of the hospitalization period. Therefore, there will be 3 groups: children born before 28 weeks of GA, infants born between 28+1 and 32 weeks of GA, and children born between 32+1 and 36+6 weeks of GA.

To be included, parents of preterm infants must meet the following criteria: (1) they must speak fluent Italian or English, (2) they must be aged ≥18 years, and (3) their children must not have other genetic pathologies or other pathologies not linked to the preterm birth.

These 3 limitations help us in creating a more consistent sample and, therefore, in obtaining more focused results. Situations such as teenage pregnancies or genetic pathologies further complicate a family’s life and, therefore, the task of assessing its well-being. While we acknowledge that they are both worthwhile topics of research, they are beyond the scope of what could reasonably be studied within the framework of our research.

To determine a satisfactory sample size for this research, we calculated using G*Power (Heinrich-Heine-Universität Düsseldorf) that the minimum sample size required, considering a probability of α error of .05, power (1 – β error probability) of 0.95, and an effect size of 0.345, will be 175 preterm infants (350 parents).

We want to stress the fact that, due to the very peculiar nature of the studied population, this will not be a probabilistic sample but rather a *convenience* sample. As explained previously, the decline in Italy’s birth rate does not allow us to use the yearly average estimation of preterm babies born in each of the partner hospitals as a reliable predictor for constructing our sample.

### Research Design

To achieve the research objectives, this study will use a mixed methods approach that combines ethnographic observations with standardized questionnaires. The goal of this approach is to enhance the results by complementing the strengths of different methodologies [30]. The quantitative aspect of this research aims to investigate the potential impact of preterm birth on the psychological well-being of parents during the early months of their child’s life. Recent literature emphasizes that preterm birth can have a significant impact on parental well-being, exposing them to a high likelihood of developing anxious, depressive, and sometimes even posttraumatic stress symptoms [31]. The questionnaires with validated scales used in this research will be used to create composite statistical indexes that measure, for each responding parent, levels of stress, depression, anxiety, perception of parental self-efficacy, quality of the bond established with their child, and the perception of the social support received. The reasons that prompted us to consider such tools stem from the literature highlighting the necessity of using specific instruments in investigating parental distress in the postpartum period, to adequately capture the specific reasons leading to the experience of such unique feelings [32]. Moreover, questionnaires with validated scales are a valuable tool in psychological research. They allow data to be collected from a large number of participants in an inexpensive and standardized manner. The ease of administration and access to personal information encourage the disclosure of candid data, reducing the social reaction effect as well as allowing people to respond more freely. The qualitative aspect of this research involves the use of the range of methods provided by ethnography, considered particularly suitable for studying the dynamics of critical health practice in action [29,33] as the ethnographic approach allows for an understanding of how medical discourse (eg, protocols, standards, and knowledge) forms within everyday medical practices (eg, informal actions, skills, behaviors, and the physician-patient relationship) that are involved in care trajectories [34]. The e-ParWelB project will use a focused ethnographic research approach with different methods. During the NICU stay, we will combine research strategies, including focused ethnography, which entails observing the relational dynamics between different staff members and between staff and parents; discussing with neonatologists their notes and accounts regarding the neonate’s care pathway; observing the habits and procedures of health care staff to reveal how prognostic knowledge is shaped; and, finally, conducting narrative interviews with the staff to capture narratives about family needs and perceptions of care practices.

**Figure 1:**
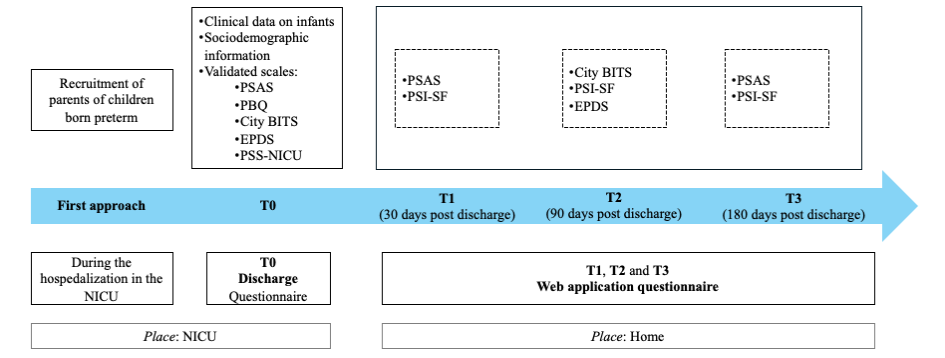
Study model. BiTS: Birth Trauma Scale; EPDS: Edinburgh Postnatal Depression Scale; NICU: neonatal intensive care unit; NPST: Nurse-Parent Support Tool; PBQ: Postpartum Bonding Questionnaire; PSAS: Postpartum Specific Anxiety Scale; PSI-SF: Parenting Stress Index–Short Form; PSS:NICU: Parental Stressor Scale: Neonatal Intensive Care Unit.

Parents will be approached and recruited by a psychologist within the NICU or sub-NICU when the medical staff announce their child’s discharge. At this stage, the project will be thoroughly explained to them, and they will be requested to provide written informed consent. Parents of premature infants will be invited to participate in this study a few days before the discharge. The timing of recruitment will be individually determined based on the assessment conducted by the neonatologist considering the infant’s prognosis. When the health care staff informs the parents that their baby will be discharged, allowing them to envision their return home, an interview will be conducted within the NICU wards (T0). During this session, a structured interview will be administered along with specially designed questionnaires combining internationally validated scales with standardized questions related to socioeconomic status (Figure 1). The administration of the self-report questionnaires will be carried out via the Qualtrics XM platform (Qualtrics International Inc; managed by the scientific coordinators of the e-ParWelB) accessible through the Qualtrics offline app using tablets.

Subsequently, the study includes a follow-up phase to monitor the psychological well-being of parents of premature infants after discharge. The follow-up will be conducted through the use of a specially designed web application accessible only to participants that will allow for the monitoring of parents’ adaptation and psychological well-being for up to 6 months after the newborn’s discharge from the NICU. Parents will be asked to complete validated scales at 3 specific time points: 30 days (T1), 90 days (T2), and 180 days (T3) after discharge from the NICU (Figure 1). The presentation order of the validated scales will vary during the 3 follow-up time points. This choice will ensure that no mechanisms of response automation based on memory are created. The responses provided by parents to the validated scales used within the web application will trigger automated alerts signaling difficult or potentially risky situations. Parents whose responses have triggered an alert will be contacted by a psychologist to offer them psychological support and possible referral to dedicated services. In addition, during this monitoring period, a series of support forums will be implemented within the e-ParWelB web application—in fact, the web application will incorporate a space for peer discussion where issues related to, for example, daily care practices or family well-being can be discussed; a private messaging channel for psychological support where each user will be able to turn to an experienced psychologist for support in case of need; and, finally, a technical forum where each user can report malfunctions or request technical support regarding the web application’s functionality.

A brief overview of the validated scales used in the surveys (in the discharge questionnaire and the follow-up questionnaires) and the topics covered by the standardized questions, which are more sociologically oriented, present in the discharge questionnaire is outlined in the following sections. Finally, a brief description of the netnography and of the focused ethnography that will take place in the NICUs and the related data collection techniques is presented.

### Measures and Instruments

#### Measures at T0 (Discharge Questionnaire)

At the time of the child’s discharge from the NICU, the investigation will focus on assessing anxiety, depressive, and posttraumatic symptomatology while also investigating parents’ perceptions of their self-efficacy in bonding with their child within the chaotic and stressful environment of the NICU.

For this purpose, several validated scales will be used. Most of the literature on postpartum anxiety [35-37] refers to measures of generalized anxiety, which can often prove problematic from a psychometric perspective [38]. To date, the only available questionnaire specifically measuring postpartum anxiety is the Postpartum Specific Anxiety Scale (PSAS) [39,40]. Furthermore, the instrument has demonstrated excellent psychometric properties (ω ranging from 0.72 to 0.90 across the 4 scales and λ4 ranging from 0.71 to 0.92 across the 4 scales). For these reasons, the research team deemed it the most sensitive and appropriate tool for capturing the construct under investigation in the target population.

Postnatal depression is the most widely measured disorder in the postpartum population, which today, in Italy, is mostly subject to routine screenings for this condition. Following a careful analysis of the most recent literature [41,42], the Edinburgh Postnatal Depression Scale (EPDS) [43,44] emerged as the most widely used self-report questionnaire for measuring postpartum depression due to its ease of administration, cultural adaptability, and excellent psychometric properties. The validated Italian version has demonstrated good validity and reliability (Cronbach α=0.7894), confirming the validity of the EPDS in identifying postnatal depression. For these reasons, the research team deemed it the most sensitive and appropriate tool for capturing the construct under investigation in the target population.

Research on posttraumatic stress disorder (PTSD) during the postpartum period has typically adapted questionnaires developed for other populations, such as war veterans, which often prove poorly suited for capturing the specificities of childbirth trauma. Perinatal research comparing general PTSD measures with specific postpartum PTSD measures has found that, although highly correlated, the agreement between these scales in identifying PTSD diagnoses is low [45]. Therefore, the choice of measurement type is crucial for identifying cases of postpartum PTSD. In addition, the criteria of the American Psychiatric Association for PTSD measurement have significantly changed in the transition from the Diagnostic and Statistical Manual of Mental Disorders, Fourth Edition, to the Diagnostic and Statistical Manual of Mental Disorders, Fifth Edition. Therefore, it is essential to adhere to these new measurement criteria. The City Birth Trauma Scale [46] is a tool specifically developed to measure childbirth-related PTSD according to the recent Diagnostic and Statistical Manual of Mental Disorders, Fifth Edition, criteria and is, to the authors’ knowledge, the only instrument with these specificities. This instrument has previously demonstrated excellent reliability (Cronbach α=0.92). For these reasons, the research team deemed it the most sensitive and appropriate tool for capturing the construct under investigation in the target population.

The Parental Stressor Scale: Neonatal Intensive Care Unit [47,48] aims to capture parents’ perception of distress experienced during their children’s stay in the NICU. To reconstruct parental distress, the underlying model of the scale considers the interaction between various psychological and physical stress factors, such as stress related to sounds, lights, noises, and other sensory experiences typical of a highly technologized health care setting [47]. The Parental Stressor Scale: Neonatal Intensive Care Unit provides the opportunity to obtain various types of information: levels of stress related to specific situations experienced by parents with preterm infants, overall stress levels, individual stressful elements, and overall tension levels. The translation and validation study in Italian has found good psychometric properties of this tool [48].

Scientific research on the complex experience of preterm birth increasingly emphasizes that such an experience can pose significant challenges for parents, particularly in the development of bonding, which is the early relationship that develops post partum between mother and child [49]. A careful analysis of the literature [50,51] allowed us to select a questionnaire widely used in investigating bonding in the postpartum period: the Postpartum Bonding Questionnaire (PBQ) [52,53], a tool designed to measure the quality of the bond between parents and children and identify whether there are disturbances in the relationship. The Italian adaptation of the PBQ was developed using the back translation procedure [54]. Studies demonstrate that the Italian version of the PBQ has good psychometric properties and can be implemented in the Italian cultural context to assess early mother-child relational difficulties [53].

Moreover, the assessment at discharge will also take into account further aspects in light of the fact that several studies have shown that some minor stress factors such as the distance from one’s home to where the child is hospitalized may contribute to a higher level of maternal discomfort [55]. The socioeconomic characteristics of family units, such as educational level, social class, job insecurity, employment status, and migration background, have also been examined as risk factors for preterm birth [56,57]. In addition, the study by Miles et al [58] revealed that the marital status of the mother is also a significant factor in predisposing to or preventing the development of a mood disorder. Specifically, married women who perceived a high level of support from their partners reported lower levels of postpartum depression than unmarried women. Conversely, among women with preterm infants, maternal age or level of education do not appear to be correlated with the onset of mood disorders [59]. Another factor that seems to mitigate the onset of mood disorders in parents of preterm infants is a high level of social support [60]. For these reasons, the discharge questionnaire will gather basic sociodemographic information from parents, such as gender, age, nationality, geographic origin, family composition, and educational level. A series of self-assessment questions will be used to evaluate the perceived social support of parents of premature infants. In particular, we have designed 6 items that inquire about perceived social support from partners, family members, friends, and health care personnel. Furthermore, respondents will be presented with a series of questions regarding the frequency of cultural activities, which will serve as indicators of their level of cultural capital, along with a series of questions aimed at capturing their occupational situation.

Finally, a validated scale will be used to gather information regarding parents’ perception of nursing support during the infant’s hospitalization, the Nurse-Parent Support Tool (NPST) [61]. Scientific research has highlighted the crucial role of parents’ perceptions of the actual support provided by nursing staff in mitigating the stressful effects of the NICU stay [62]. In light of this evidence, Miles et al [61] devised the NPST, a scale specifically addressing the educational and informational aspects of staff support as perceived by parents that is widely used in NICUs today [63]. The NPST is a 5-point Likert-scale questionnaire consisting of 21 items divided into 4 groups: informational support, emotional support, appraisal and parental esteem support, and caregiving support. Each item is rated on a scale from 1 (*Almost never*) to 5 (*Almost always*), with higher scores indicating a higher perceived level of support provided by the nursing staff. The validity and reliability of the Italian version of the NPST were assessed by Montirosso et al [48].

#### Follow-Up Measures at T1, T2, and T3 (Web Application Questionnaire)

At the time of discharge, the involved parents will be invited to register on the web application specially designed for this research. Through the web application, participants will independently complete 3 short questionnaires at the 3 different times designed for follow-up (T1 at 30 days after discharge of the newborn, T2 at 90 days after discharge of the newborn, and T3 at 180 days after discharge of the newborn) to monitor their mental health status. In the web application, some validated scales already proposed at the time of discharge will be implemented. The scales are presented to participants in different moments to minimize potential bias due to response based on learning or recollection. Specifically, (1) *during T1*, participants will be invited to independently complete the PSAS and the Parenting Stress Index–Short Form (PSI-SF); (2) *during T2*, participants will be invited to independently complete the City Birth Trauma Scale, the EPDS, and the PSI-SF; and (3) *during T3*, participants will be invited to independently complete the PSAS and the PSI-SF.

The only validated scale for which completion will be required at every follow-up time point is the PSI-SF [64,65]. The existing literature has highlighted the possibility of early identification of stressful parent-child systems to develop interventions aimed at reducing stress that could decrease the frequency and intensity of emotional and behavioral disorders in children in our society [65,66]. The characteristics of the child, those of the parent, the family context, and particularly stressful life events are some of the elements of the parent-child system that have been identified as important [64,67]. The Parenting Stress Index was developed in response to the need for an assessment measure of these characteristics. For the purposes of this scientific research, the PSI-SF was adopted, which is an abbreviated version of the Parenting Stress Index that investigates parental distress, dysfunctional parent-child interactions, and difficult child behaviors. The brevity of the PSI-SF allows primary health care providers to identify families most in need of follow-up services [68]. Furthermore, the PSI-SF represents an extremely interesting, agile, easy-to-administer, and interpretable tool. The Italian version of the PSI-SF has been translated and validated by Guarino et al [69] and investigates stressful systems through 3 subscales: parental distress, dysfunctional parent-child interaction, and difficult child behaviors.

Our intention is to use the PSI-SF as a potential *risk screening tool*, namely, a technique that can identify parent-child systems under excessive stress and, thus, serve as an important component within prevention programs aimed at reducing the frequency and intensity of emotional and behavioral disorders [69]. The responses provided by parents to all validated scales used within the web application, in cases in which they exceed the threshold values established for each scale, will indeed allow for the automated activation of alert signals for situations of difficulty or potential risk. Finally, as mentioned previously, all psychological scales used in this study are internationally validated, ensuring their reliability across diverse populations, including those with preterm infants. The sociodemographic section of the questionnaire was tested in a pilot study involving 104 parents [12], which preceded this project and allowed for refinement of the questions to better suit the target population. While these tools provide a strong foundation for data collection, future research could explore additional validations to further tailor the measures to this specific group.

### Netnography

As mentioned previously, the web application will feature three specific communication channels through which users can interact: (1) a public forum for peer discussion only where users will be able to post messages, share opinions and ideas, or ask questions on issues related to the daily childcare at home; (2) a private communication channel through which users can contact the psychologist to ask for support in case of discomfort, uneasiness, or other states of distress; and (3) a forum dedicated to the technological tools available to participants in which it is possible to both report technical malfunctions to IT staff and discuss among parents and researchers the impacts of the eHealth tool on its users and their well-being.

The textual corpora produced by users will be downloaded and will form the empirical basis for data analysis through an approach of digital ethnography.

For many years now, computer-mediated communication has been fully integrated into people’s daily lives, blurring the distinction between offline and web-based social worlds as they both coexist and fully overlap [70]. Therefore, it has become increasingly important and relevant for social researchers to study society in and through the internet. Although methodological reflections around digital research have been manifold and have also given rise to different terminologies and definitions, in this contribution, we use the term *netnography*, coined by Kozinets [71], to illustrate our digital ethnography approach that adapts the typical techniques and tools of ethnography to the study of web-based communities, which interact through a computer-mediated communication. The presence of the researchers, as well as the fact that messages written in the forums will be subject to analysis, is clear to the participants from the outset, so our netnography will use an overt observation technique.

In our project, the web-based community is closed, meaning that the web application and its tools can only be accessed through a username and password if one participates in the study and has agreed to take part in the follow-up phase. Some methodological perspectives on web-based research place great importance on the medium [72] and, by consequence, on the specific digital environment and its characteristics that shape the spaces, times, opportunities, and constraints under which web-based interactions take place. In our case, one of the main constraints is that messages in the public forums (*n. 1*, a public forum for peer discussion, and *n. 3*, a forum dedicated to the technological tools) will be published subject to approval by the members of the research team to avoid the use of inappropriate language or extremely off-topic communications. If some messages are rejected, they will also end up in the analysis, but they will obviously not affect the overall interaction as they will not be visible to other users. Time is another important feature that will have consequences for the flow and volume of communications as the web application will see the asynchronous access of participants, who are gradually recruited over time. Although the messages in the public forums will remain fixed and always visible, participants who complete the 180-day follow-up period may have less incentive to access the web application, resulting in no further discussion with other newly recruited participants. On the other hand, the accumulation of messages over time could stimulate other interactions, also providing an incentive for participants to use the web application not only as a tool for filling out periodic questionnaires. From a research point of view, we are particularly interested in the volume, modalities, and content of the digital interactions between participants, aware of the constraints to which such communications are subjected. It will be useful to understand the representations, perceptions, and perspectives of premature parents and the impact of prematurity and of the care practices in daily life. Moreover, we will pay attention to the adaptation processes carried out to cope with the transition period after discharge from the NICU and how parents deal with their new domestic life. In the analysis, even though the participants are pseudoanonymized through a unique alphanumeric code, we can trace and use as a segmentation variable the one that discriminates between the partner who gave birth and the one who did not, for example, to account for differences in the prevalence and occurrence of the messages and topics reported. Furthermore, the *n. 3* forum has the goal to explore how digital-driven changes affect families. It is dedicated to specifically discussing the tools in the web application through a process of facilitation by a member of the research team stimulating dialogue on dedicated questions. As previously stated, this section of discussion about the web application is intended not only as technical support but also as a space for group reflection on the impact of the digital tools introduced by e-ParWelB to support families’ well-being. The material created by users in this section will then be analyzed with the aim to explore whether web-assisted alerts and expert and peer support on the web were beneficial to families’ well-being and, eventually, how these tools might be improved in the future. This phase of research will also allow us to reflect upon the implications of introducing a digital medium in the relationship between the health care staff and families [73], as in the tradition of sociology and science and technology studies working together on health care issues [74].

### Ethnography

The qualitative part of this research aims to study the NICU environment through the use of focused ethnography [75,76]. This term indicates a research method based on brief and intensive observations with the aim of analyzing well-defined phenomena within a specific context. While the classic form of ethnography is centered on the study of broad topics, such as the holistic observation of social groups or institutions, focused ethnographies narrow down their perspective to the analysis of specific actions, interactions, and social situations. This implies a few changes in the position of the observer compared to the situation of traditional ethnography. First, the researcher cannot face the fieldwork unprepared but must be at least theoretically confident with the context of the actions that are to be studied. Second, traditional ethnography provides a range of observer positions, from participant as observer to observer as participant [77], whereas focused ethnography does not involve the same opportunity for observing as a participant because of the nature of the object of study. It would be both highly inappropriate and technically impossible for the focused ethnographer to participate actively in, for example, a medical examination or an operation. Instead, a more distant observer position is possible. In this way, the focused ethnography researcher may be excluded from contextual factors of importance. While traditional ethnography affords the chance to actually participate in the life of the studied social milieu, focused ethnography does not offer the same opportunities of active participation. This usually happens due to the inherent characteristics of the participants under investigation. Active participation in skill-intensive activities would be both highly unrespectful and unfeasible for the focused ethnographer. Consequently, it is said that, in focused ethnography, researchers are not doing observant participation but that they are participating as observers.

A brief recap of the main traits of focused ethnography and its difference from traditional ethnography is shown in Table 1.

**Table 1:** Comparison between focused ethnography and traditional ethnography derived from the study by Andreassen et al [78].

Characteristic	Focused ethnography	Traditional ethnography
Subject matter	Episodes in social fields Clear research focus Familiar cultures Background knowledge before data collection Applied research	Entire social fields Broad research purpose Foreign cultures Gaining knowledge from engagement in the field Basic research
Data collection	Relatively long planning phase Intermittent visits with particular time frames Focused exploration Video or audio recordings or detailed, focused field notes Often multi-sited Time intensity	Relatively short planning phase Full-time participant observation over a longer period Open exploration Extensive and in-depth written field notes Often single sited Time extensity
Researcher role	Alterity Observer as participant Selected informants who hold a specific knowledge serve as key participants	Strangeness Participant as observer Participants are often those with whom the researcher develops close relationships
Data analysis	Analysis intensity Collective data analysis sessions	Experiential intensity Solitary data analysis

Focused ethnography is a particularly appropriate technique for our research due to the traits of medical practice, which comprise bounded and clearly delineated social occurrences or scripted exchanges. This statement has been proved true by an increasing number of studies using this technique within the hospital setting in recent years [79-82]. This technique affords the means to investigate particular episodes or interactions within social milieux such as NICUs, facilitating nuanced and comprehensive insights into the influence of sociocultural factors on the interaction between the NICU staff and preterm parents. This specific effort of focused ethnography will be divided into 2 steps, each involving the staff members of NICUs in hospitals collaborating with the project. First, the researcher will observe the routine practices that constitute what is defined as *ward life*, trying to create as little interference as possible with the staff’s activities. At this stage, the researcher’s focus will not be on individuals but rather on the overall context of neonatal intensive care and the social interactions occurring within it. Second, the researcher will use narrative interviews to gather additional information from the NICU staff. The use of interviews is quite common in focused ethnography, as explained by the relevant literature on the topic: “The focused ethnography researcher may be precluded from contextual factors of importance. Applying method triangulation as a cornerstone in ethnography is a way of overcoming this dilemma. For example, combining short observations with interviews will give opportunities to ask about the context in which the observations take place, as well as to explore how the participants experience being observed” [78]. In these narrative interviews, the researcher will aim to involve staff members with profiles as diverse as possible regarding their roles, length of service, gender, and age. The objective is to achieve what the literature calls a “maximum variation sample” [78], maximizing sample diversity (while acknowledging that this is a nonprobabilistic convenience sample) across certain characteristics to capture the complexity of the studied reality.

Throughout both of the aforementioned steps, this research will try to analyze the meaning and effects of the NICU staff decisions and practices on the parents’ well-being. This focus on the analysis of existing procedures in skill-intensive contexts, especially medical-related ones, has been referred to in previous research as *exnovation*. *Exnovation* refers to the attempt to foreground what is already present—though hidden—in specific practices to render explicit what is implicit in them [83]. A focus on exnovation allows us to bring to light implicit matters of actual practice and develop a fresh perspective on the ingenuity of the professionals and the specific structure of their practices. It offers insights into their specific modes of ordering day-to-day practices [84]. Exnovation, in other words, elucidates competencies of coordination and alignment of these modes of ordering of which those involved are not always aware [29]. In other words, this research aims to reflect on existing practices of care aimed at preterm parents and their children and deepen our understanding regarding their effects with the aim of preventing family maladjustment after a traumatic event such as preterm childbirth.

Regarding the organization of the qualitative tools for the research, the access to the field will be negotiated separately in each NICU to take measures to avoid disrupting staff work and minimize impact on organizational routines. To conduct an ethical and respectful data-gathering process, NICU staff will be informed about the research in advance by the department heads, with whom a partnership has been established for the project, thus ensuring that they are aware of the research procedures. Once access is granted, the researcher present in the department will autonomously organize interviews with the staff.

### Expected Outcomes

The e-ParWelB central impact will be developing and piloting a model of sociopsychological assessment tailored for parents whose infants have been hospitalized in the NICU for critical health situations connected to prematurity. The model, by increasing preterm parents’ perception of self-efficacy and well-being, will allow for the prevention of family maladjustment [5,6] and, therefore, the indirect improvement of preterm child health outcomes [7]. The mixed methods social research that we designed is expected to achieve the following results: (1) to reduce the discomfort and maladjustment caused by prematurity on preterm parents and assess their perceptions of social support (from family, friends, potential employers, and colleagues), perceptions of preparedness, and parental self-efficacy from the time of discharge and for the following 6 months; (2) to analyze the difference between mothers’ and partners’ responses to preterm birth in terms of stress, negative feelings, and perceptions of parental self-efficacy and social support; (3) to determine which neonatal care practices, forms of communication, and environmental settings of the NICU are more likely to reduce negative feelings and foster the well-being of preterm parents; and (4) to advance the knowledge on the role of a digital-based tool in monitoring and supporting families’ well-being during the follow-up.

On the latter point, e-ParWelB intends to engage in the relationships among science, technology, and society by addressing how the hospitalization experience of preterm families may inform technology and science development. At the same time, we aim to identify the effect of the introduction of a digital technology–driven assessment and support model on families’ well-being and on care practices in the neonatal intensive care wards. In particular, the e-ParWelB project directly connects with the objectives of (1) developing a public health system enhancing investments in terms of human, digital, structural, instrumental, and technological resources; and (2) improving scientific research in the biomedical and health field.

Indeed, the interdisciplinary approach to promote eHealth in premature families enhances the dialogue between science (health care and medical knowledge) and society (families’ perspectives) on the topic of preterm birth through the aid of digital technology. This will be possible with the implementation of the e-ParWelB web application, which will follow processes of responsiveness and adaptation as well as considering criteria of accessibility following responsible research and innovation principles. As the web application constitutes both a tool to periodically monitor parents’ well-being and a digital space for sharing and discussion, both with a psychologist and among peers, the project has a multi-side technological and scientific impact.

At the same time, we aim to identify the effect of the introduction of digital technology on families’ well-being and on care practices. For instance, through the netnographic exploration of the web-based space of discussion within the e-ParWelB web application, we aim to address the eventual health care web-based community of practice forming in the forum [85]. Drawing on the idea by Timmermans and Berg [86], an orientation of *technology in practice* allows for a critique by scholars in the social sciences regarding the complicated modalities in which the *social* and the *material* intertwine in technologies for health care, as well as possibly influencing their creation and implementation. Thus, referring to what in science and technology studies literature is known as the *sociotechnical approach*, we are interested in how the social and health care aspects influence the development of the digital technology and, at the same time, in modalities through which the digital medium affects families and the care process, especially after discharge [87,88]. Therefore, we also aim to understand the impact of the web application on families’ well-being.

## Results

We expect to observe these results by the end of October 2025, the date set as the conclusion of the Project of Relevant National Interest funded by the Ministry of University and Research.

## Discussion

### Impact

This research project aims to identify the social determinants of preterm parents’ well-being. It will monitor their feelings and perceptions of well-being during the baby’s hospitalization and after discharge, with the goal of reducing their distress and discomfort. Unlike previous studies, this project places a significant emphasis on both mothers and fathers, analyzing how gender differences and parental roles influence their experiences and affect outcomes related to perceived well-being and social support. Furthermore, using ethnographic methods, this study delves into the everyday life of NICU wards. It examines these environments both as sites of high-skill professional practice and as arenas of social interaction between staff and parents. The aim is to understand the social arrangements and practices that facilitate staff work and parental participation and involvement during the sensitive period of their baby’s hospitalization. Finally, the use of technology in this study serves a dual purpose. Instrumentally, it enables the monitoring of parental well-being after discharge, but it also prompts deeper sociological inquiries into its role at the intersection of medical science and society. In addition, technology acts as a medium for mutual aid, fostering specific web-based peer discussions and shaping new forms of social support and community among parents of preterm infants.

The e-ParWelB project not only contributes to scientific and technological development, but its impact on the social and economic dimensions also stems from the premise that, by increasing the well-being of preterm parents it is possible to indirectly improve their infants’ health outcomes. As scholars have argued, the abrupt disruption of the establishment and development of parental mental representations, combined with the possibility that both the baby and the mother are in critical condition, can make preterm birth a traumatic event for parents [89]. Therefore, reducing the family’s maladjustment enhances future health—including mental health—in premature children [7]. Moreover, parents’ mental health is supported, as well as their self-efficiency, preventing a large set of repercussions. For society at large, this also means introducing factors of prevention in terms of social and economic costs that unhealthy individuals and families might incur. Furthermore, the situation of premature birth care in Italy sees scattered NICUs applying different protocols for what concerns the role played by the parents of hospitalized newborns. As the latest report of the Italian Society of Neonatology shows, for instance, just 63% of the NICUs allow free-time access for parents [90]. When looking at the regional location, we observe an unequal distribution of the free-time access wards. Indeed, in the northern regions, 88% of NICUs do grant free-time access, whereas in the south, just 34% percent do [90]. Thus, by developing a piloted model for preterm parents’ sociopsychological assessment, e-ParWelB will produce knowledge about the best practices to be implemented in NICUs, enhancing the opportunity for stakeholders and policy makers to make neonatal care protocols less regionally uneven and more efficient when concerning the collaboration between parents and health care staff.

### Strengths and Limitations

The e-ParWelB project was preceded by the ParWelB project (principal investigator: AD), and although with a different institutional setup and collaborations with different NICUs, the 2 projects stand in a relationship of continuity, building on the same theoretical and methodological premises. Therefore, practical experiences and careful analyses regarding actual strengths and limitations have already been carried out for the previous project [10].

In general, the greatest strength of the entire research project lies in listening to and empowering the parents of premature children who participate in the study, making them more aware of and helping them deal with the challenging situation of prematurity, advancing deep reflections that do not stop at purely medical practices but also promote a person-centered approach to stimulate new neonatal practices and foster the well-being of families.

In this context, it is essential to reconnect the social dimension with the medical experience, also owing to the support of technology, and this is one of the main objectives of the project.

Indeed, we aim to study a model for monitoring and assessing parental well-being and look at the relationship between the medical-nursing staff and the parents of premature babies from a sociological and psychological perspective with the aim of promoting public awareness and participation, also by involving the medical-nursing staff with respect to the values, personal experiences, and point of view of the parents.

These strengths are accompanied by deep reflection in mapping out the potential limitations of the project and strategies to mitigate them.

Among these, access to the study is worthy of consideration. An actual constraint on access for preterm parents is language—the lack of cultural mediators and translators in the project allows only for participation of people fluent in Italian and English. As Italian and English were also the only languages in the previous ParWelB project, we have already ascertained that a nonnegligible proportion of parents, often with migration backgrounds, were unable to participate in the study, and we expect this situation to be repeated. This is uncomfortable for mainly 2 reasons. First, it does not allow a specific population of parents, who often are more likely to be in situations of marginality or social isolation, to access the direct and indirect benefits of the project, such as stimulating self-reflections on their parental role and gaining access to psychological support after discharge. Furthermore, from a purely scientific point of view, there is less opportunity to investigate how certain cultural differences and social support (which is often lacking or expressed in different ways among people with a migratory background) impact the well-being of preterm parents and their experiences in the NICU.

One of the main aspects to consider when reflecting upon the pros and cons of this project is the possible bias introduced by the implementation of the web application, which is a digital technology not necessarily available to all social groups. Digital literacy, availability of connection to the internet, and access to digital devices are all necessary conditions to participate in the study, and the lack of one of these aspects may act as an additional exclusion factor, possibly affecting marginalized social groups or groups with specific needs [91,92]. Nonetheless, parents excluded from the digital space are necessarily excluded from tools of expert and peer support.

In addition to access barriers to the study, this project faces other possible limitations regarding engagement and participation during the research activity that can be reversed. In the previous ParWelB project, due to the COVID-19 pandemic, researcher access to the NICU was restricted, so parent recruitment, discharge survey administration, and web application registration were performed entirely by NICU staff, usually by psychologists or neonatologists. Overall, in this way, NICU staff were more involved, but some methodological forms of control by the researchers were lacking regarding modalities of both study presentation and survey administration to parents [93]. In the new e-ParWelB project, while we no longer have the limitations of the pandemic, a blended approach was chosen as we will maintain a deep involvement by NICU staff, especially psychologists, in recruitment, survey administration, and web application enrollment at discharge. Above all, previous experience has shown that the relationship of trust built between the NICU staff and parents is central to engaging the latter in the project. Therefore, it is in the project’s interest to maintain these relationships both to provide parents with referents whom they consider reliable and with whom they have already become familiar during their stay in the NICU and to engage the medical-nursing staff further, stimulating an even closer relationship between these 2 social actors beyond purely medical practice. On the other hand, in the previous project, the participation rate dropped by 10% for parents recruited in the second half of the project timeline, an indicator that the motivation and consequent convincing power of the NICU staff toward parents slightly declined over time [93]. Therefore, the presence of a researcher can also be decisive in rekindling motivation and emphasizing the importance of parental participation throughout the recruitment phase. In the e-ParWelB project, in some NICUs, there will be a constant presence of a project researcher, who will take part in recruitment together with the staff and will personally take care of survey administration at discharge and invite participants to register on the web application. In NICUs where the constant presence of the researcher is not expected, it will be arranged if no one in the NICU can recruit parents or the research team notices a decline in the participation rate. Therefore, this flexible choice allows us to maintain and strengthen the bond between the parents and the NICU staff and, at the same time, provide help and support to the participants or NICU staff and ensure the correct conduct of the research from a methodological and ethical point of view.

In conclusion, one of the critical aspects of the ParWelB project was the high dropout rate during the follow-up surveys using the web application. To address this issue, the new project, e-ParWelB, proposes a lighter monitoring approach, with a shorter total period and lower frequency (6 months of monitoring instead of 1 year and 3 surveys instead of 12), thus impacting participants to a lesser extent. A less intrusive follow-up, both in terms of frequency and volume of questionnaire completion, will not affect the capacity of researchers to monitor the well-being of the participants, whereas it will lower the commitment required from users, allowing them to allocate their time and energy to other activities related to the web application, such as forums.

## Appendix

Multimedia Appendix 1 Peer review report from the Italian Ministry of University and Scientific Research - Project of National Relevance - Bando 2022 / Ministero dell'Università e della Ricerca - Direzione Generale della Ricerca del MUR (Italy).

## Abbreviations

e-ParWelB: eHealth Study on Preterm Parents’ Well-Being
EPDS: Edinburgh Postnatal Depression Scale
FICare: Family Integrated Care
GA: gestational age
NICU: neonatal intensive care unit
NPST: Nurse-Parent Support Tool
ParWelB: Study on Preterm Parents’ Well-Being
PBQ: Postpartum Bonding Questionnaire
PSAS: Postpartum Specific Anxiety Scale
PSI-SF: Parenting Stress Index–Short Form
PTSD: posttraumatic stress disorder

## Data Availability

The datasets generated or analyzed during this study are not publicly available due to the inclusion of sensitive information, such as respondents’ psychological distress, diagnosis of psychopathology, and clinical data on infants admitted to the neonatal intensive care unit. However, deidentified data may be made available from the corresponding author on reasonable request in accordance with ethical and legal considerations.
