# Supplementary material for: Preventing Premature Family Maladjustment: Protocol for a Multidisciplinary eHealth Study on Preterm Parents’ Well-Being
Source: JMIR Res Protoc. 2025 Mar 18;14:e63483. doi: 10.2196/63483 (PMC11962333; doi:10.2196/63483)
Supplement: Multimedia Appendix 1 [file resprot_v14i1e63483_app1.pdf]

Valutazione

Project code: **20225R7XB3**

Project title: **Preventing Premature Family Maladjustment: an e-Health Interdisciplinary Research (e-ParWelB)**

Coordinator: **Alessandra DECATALDO** ERC: **SH3_14**

University: **Università degli Studi di MILANO-BICOCCA**

Evaluation Summary Report

1. Quality of the research project - scientific merit and innovative nature of the project from an international perspective

CRITERIA	EVALUATION
a) Clarity and originality of the project objectives	*(provide a score from 1 to 10)* **10**
b) Relevance of the proposed project as to the specific scientific area	*(provide a score from 1 to 10)* **10**
c) Coherence as to the methodology adopted in terms of the project structure and the relative objectives with specific reference as to the contribution of the local units	*(provide a score from 1 to 10)* **10**
d) Positioning of the project as to the state of the art in the specific scientific area	*(provide a score from 1 to 10)* **10**

The overall soundness of the proposal is duly justified. The project explores several impactful theoretical arguments, and their links in the empirical study are well explained, thus making the key points of the project easy to assess. The study design and methodology are cohesive, aligned with the intended objectives, and explained with the proper level of detail. Information about participants' recruitment is well detailed, as well as inclusion and exclusion criteria. Reference to mitigation risks is solid enough. The link between goals and intended outcomes is cohesive, and again well explained. There are no weak points to highlight.

With reference to the score awarded, please provide the relative motivations by answering tothe following questions:

1. *To what extent are the project objectives structured in a clear and original manner?*
2. *What are the key points of the proposed project as to the specific scientific area?*
3. *In what way are the adopted project methodology and objectives, with specific reference as to the contribution of the local units, coherent with the project goals?*
4. *To what extent do the objectives go beyond the state of the art in the specific scientific area?*

2. Composition of the research team, feasibility and appropriateness of the project – scientific merit of the research team, feasibility of the work plan and appropriateness of the funding request

CRITERIA	EVALUATION
a) Expertise of the Principal Investigator, the heads of the local units and the research team	*(provide a score from 1 to 10)* **10**
b) Ability as to the implementation of the proposed project (qualification, composition and complementarity of the team)	*(provide a score from 1 to 10)* **10**

c) Organisation of the project as to the proposed objectives, the timeframe considered necessary to complete the project and the resources required (consumables, equipment, management)	*(provide a score from 1 to 10)*<br>**8**
d) Consistency of the time commitments of the members of the research team, appropriateness and relevance of the spending plan as to the objectives and time distribution of the activities	*(provide a score from 1 to 10)*<br>**7**

The PI and research team show previous experience in research activities related to their aims in this project and possess complementary expertise to ensure the successful implementation of the different stages of the project. The timeframe is feasible, and the need of resources is well described. The allocation of person*months to tasks would have improved the understanding of costs allocation. This flaw limits the overall assessment of the consistency of the time commitments of members of the research team, appropriateness and relevance of the spending plan as to the objectives and time distribution of the activities.	*With reference to the score awarded , please provide the relative motivations by answering to the following questions:*<br>*1. To what extent have the PI, the local unit heads and the research team demonstrated their scientific expertise?*<br>*2. To what extent is the project feasible and coherent in terms of competence, composition, and complementarity of the research team?*<br>*3. To what extent is the project consistent with the proposed objectives, the timeframe considered necessary for its completion and the resources required (consumables, equipment, management)?*<br>*4. To what extent is the time commitment of the members of the research team consistent with the objectives and activities of the project? To what extent is the spending plan coherent and relevant to the objectives and activities of the project)?*

3.Project Impact

CRITERIA	EVALUATION
Assessed on the basis of one or more of the following criteria:<br>• advancement of knowledge<br>• technological innovation and/or industrial applications<br>• compliance with the principle of Do Not Significant Harm (DNSH)<br>• scientific community and its strengthening<br>• internationalisation of Italian research<br>• social welfare and/or cultural developmen<br>• dissemination/sharing of scientific knowledge	*(provide a score from 1 to 20)*<br>**20**

The project contributes to advance knowledge; issues regarding the measure of challenges that research faces in terms of technological innovation and industrial applications do not apply to this application. The project respect the principle of Do Not Significant Harm, and is likely to have an impact on the scientific community through several complementary dissemination strategies, including fostering international research collaborations. Therefore, the results not only build on previous research but also are likely to contribute to the internationalization, and to social welfare and/or cultural development.	*As to the awarded scoring, kindly provide the relative motivations by answering, also aggregately, the following questions:*<br>*1. Does the project advance knowledge?*<br>*2. Does the project measure up to the challenges that research faces in terms of technological innovation and industrial applications?*<br>*3. Does the project respect the principle of Do Not Significant Harm (DNSH)?*<br>*4. Will the project have an impact on the scientific community? How will it strengthen it?*<br>*5. Will the project increase the internationalisation of Italian research?*<br>*6. Will the project contribute to social welfare and/or cultural development?*<br>*7. Does the project propose actions to disseminate knowledge and its results?*

TOTAL SCORE **95**

© 2024 - MUR - Direzione Generale della Ricerca - in collaborazione con CINECA v.1.0.0.0
